# Patient and Staff Perspectives on the Impacts and Challenges of Hospital-Based Harm Reduction

**DOI:** 10.1001/jamanetworkopen.2024.0229

**Published:** 2024-02-22

**Authors:** Leah Fraimow-Wong, Marlene Martín, Laura Thomas, Ro Giuliano, Oanh Kieu Nguyen, Kelly Knight, Leslie W. Suen

**Affiliations:** 1 University of California, San Francisco, School of Medicine; 2 Division of Hospital Medicine at San Francisco General Hospital, University of California, San Francisco; 3 San Francisco AIDS Foundation, San Francisco, California; 4 Department of Humanities and Social Sciences, University of California San Francisco; 5 Division of General Internal Medicine at San Francisco General Hospital, University of California, San Francisco

## Abstract

**Importance:**

Harm reduction is associated with improved health outcomes among people who use substances. As overdose deaths persist, hospitals are recognizing the need for harm reduction services; however, little is known about the outcomes of hospital-based harm reduction for patients and staff.

**Objective:**

To evaluate patient and staff perspectives on the impact and challenges of a hospital-based harm reduction program offering safer use education and supplies at discharge.

**Design, Setting, and Participants:**

This qualitative study consisted of 40-minute semistructured interviews with hospitalized patients receiving harm reduction services and hospital staff at an urban, safety-net hospital in California from October 2022 to March 2023. Purposive sampling allowed inclusion of diverse patient racial and ethnic identities, substance use disorders (SUDs), and staff roles.

**Exposure:**

Receipt of harm reduction education and/or supplies (eg, syringes, pipes, naloxone, and test strips) from an addiction consult team, or providing care for patients receiving these services.

**Main Outcomes and Measures:**

Interviews were analyzed using thematic analysis to identify key themes.

**Results:**

A total of 40 participants completed interviews, including 20 patients (mean [SD] age, 43 [13] years; 1 American Indian or Alaska Native [5%], 1 Asian and Pacific Islander [5%], 6 Black [30%]; 6 Latine [30%]; and 6 White [30%]) and 20 staff (mean [SD] age 37 [8] years). Patients were diagnosed with a variety of SUDs (7 patients with opioid and stimulant use disorder [35%]; 7 patients with stimulant use disorder [35%]; 3 patients with opioid use disorder [15%]; and 3 patients with alcohol use disorder [15%]). A total of 3 themes were identified; respondents reported that harm reduction programs (1) expanded access to harm reduction education and supplies, particularly for ethnically and racially minoritized populations; (2) built trust by improving the patient care experience and increasing engagement; and (3) catalyzed culture change by helping destigmatize care for individuals who planned to continue using substances and increasing staff fulfillment. Black and Latine patients, those who primarily used stimulants, and those with limited English proficiency (LEP) reported learning new harm reduction strategies. Program challenges included hesitancy regarding regulations, limited SUD education among staff, remaining stigma, and the need for careful assessment of patient goals.

**Conclusions and Relevance:**

In this qualitative study, patients and staff believed that integrating harm reduction services into hospital care increased access for populations unfamiliar with harm reduction, improved trust, and reduced stigma. These findings suggest that efforts to increase access to harm reduction services for Black, Latine, and LEP populations, including those who use stimulants, are especially needed.

## Key Points

**Question:**

What are the perspectives of patients and staff on the impacts and challenges of hospital-based harm reduction services?

**Findings:**

In this qualitative study that included semistructured interviews with 20 hospitalized patients receiving harm reduction services and 20 staff serving such patients, major themes included expanded access to harm reduction services for structurally marginalized patients, improved trust between patients and staff, and decreased staff stigma toward patients who use substances. Challenges included staff hesitancy regarding regulations and limited prior substance use education among staff.

**Meaning:**

These findings suggest that, amidst a continuing overdose crisis, providing hospital-based harm reduction services may expand access to critical interventions and positively improve hospital care.

## Teaser

This qualitative study analyzes patient and staff perspectives on a hospital-based harm reduction program for patients with substance use disorders in California.

## Introduction

Annual US deaths from drug-related overdose have doubled in less than a decade and substance use disorders (SUDs) account for 1 of 9 hospitalizations.^1,2^ Although hospitals spend $13.2 billion yearly caring for patients with SUDs, addiction treatment access remains low.^3,4^ Prior qualitative work on hospital addiction care^5,6,7,8^ has highlighted pervasive stigma and frequent mismatch between patient and clinician goals. Consequences of such stigma include increased self-directed discharge, avoidance of medical care, decreased retention in treatment, and increased substance use.^7,9,10^

Harm reduction, an approach developed by people who use drugs, may help bridge these gaps.^11^ Harm reduction includes provision of safer use supplies (eg, syringes, pipes, fentanyl test strips, and naloxone), education, and destigmatized care that promotes health anywhere on the continuum of substance use—contrasting with abstinence-only frameworks.^11^ Harm reduction decreases overdose mortality, increases uptake of medication treatment, and engages people with SUDs in care.^12,13,14,15^

Canadian hospitals have been forerunners in integrating harm reduction services into hospital care, finding improved mental health and decreased substance use.^16,17,18,19^ Several US hospitals and ambulatory clinics have followed suit, with growing interest from health systems nationwide as overdose deaths increase.^20,21,22,23^ However, data remain limited on patient experiences with these services in the hospital, and, to our knowledge, no studies have examined both patient and staff perspectives on US, hospital-based harm reduction.

Since 2020, our urban, safety-net hospital has offered harm reduction education and supplies through our interprofessional Addiction Care Team (ACT) partnership with a local syringe service provider (SSP). Primary teams consult ACT for SUD diagnosis, medication treatment, harm reduction services, and linkage to addiction care.^24^ Interested patients receive harm reduction supplies at discharge, and ACT members educate staff about harm reduction services provided. We performed a qualitative evaluation of these services, with the goal of building a model explaining the perspectives of hospital-based harm reduction for patients and staff, understanding the program’s challenges, and developing recommendations to inform clinical practice and implementation nationwide.

## Methods

### Participant Recruitment

This qualitative study was approved by the University of California, San Francisco institutional review board and followed the Consolidated Criteria for Reporting Qualitative Research (COREQ) reporting guideline to ensure rigor.^25^ We recruited participants from an academic, safety-net hospital in San Francisco, California that serves a racially, ethnically, and linguistically diverse patient population. We interviewed hospital staff and hospitalized adults with SUD aged 18 and older who were seen by ACT and received harm reduction education and/or supplies. We included patients with alcohol use disorder because they are included in our harm reduction services (eg, education on safer drinking plans and supervised withdrawal).^24^ Eligible staff included ACT and non-ACT clinicians, nurses, and navigators caring for patients receiving harm reduction services. We used purposive sampling to recruit patients with diverse racial and ethnic identities, language, and SUDs; and staff with varied hospital roles, departments, and perspectives on harm reduction. Findings attributed to staff reflect sentiments expressed by both ACT and non-ACT staff, unless otherwise denoted. Participants provided oral informed consent and received $50 for participating; written informed consent was waived by the institutional review board in accordance with the Common Rule to preserve the anonymity of the participants.

### Qualitative Interview Procedures

Two researchers (L.W.S. and M.M.) drafted 2 interview guides (1 for patients and 1 for staff) to assess perspectives on impact, facilitators, and barriers for harm reduction services, with the goal of developing a conceptual framework. Study coinvestigators included community partners who provide SUD services (L.T. and R.G.) and other content experts (K.K. and O.K.N.). All investigators provided interview guide feedback. Finalized guides focused on patient and staff experiences with receiving and delivering harm reduction services, perspectives of the impact on care quality and stigma, prior experiences with harm reduction, program challenges, and recommendations for improvement, along with demographic data including self-reported race and ethnicity and hospital role (eAppendix 1 in Supplement 1). Self-reported race and ethnicity categories included American Indian or Alaska Native, Asian and Pacific Islander, Black, Latine, White, and multiracial; race and ethnicity were included to ensure diversity of perspectives. The researchers (L.F.W. and L.W.S.) used these guides to conduct semistructured interviews in English or Spanish between October 2022 and March 2023 until data saturation was met. Interviews lasted a mean (range) of 40 (16-65) minutes and occurred in person or by video conferencing (Zoom Video Communications). Interviewer L.F.W. was a 34-year-old Asian and White cisgender woman in medical school who previously worked as a community health worker and researcher; L.W.S. was a 35-year-old Asian cisgender woman working as an addiction medicine physician and health services researcher; neither identify as having personal lived experience with SUD.

### Data Analysis

We audio-recorded, professionally transcribed, and coded interviews using Dedoose version D 9.0.85 (SocioCultural Research Consultants).^26^ We analyzed the data using thematic analysis, with data collection and analysis occurring simultaneously.^27^ L.F.W. and L.W.S developed the preliminary codebook by reviewing interview recordings, transcripts, and analytic memos. L.F.W. and L.W.S. independently coded 2 interviews, applying deductive coding from the preliminary codebook and updating the codebook with inductive codes drawn from the data. They repeated the process for 2 more interviews until reaching consensus, and L.F.W. coded the remaining interviews, iteratively, updating the codebook throughout (eAppendix 2 in Supplement 1). All authors reviewed preliminary findings and met regularly to discuss themes, the conceptual model, and recommendations, reconciling differences until consensus was met. Following the Morse distinction between themes and categories in qualitative analysis, we reviewed the categories that emerged as relevant to program challenges.^28^ Participant (P) and staff (S) numbers accompany all quotations.

## Results

We interviewed 20 patients (mean [SD] age, 43 [13] years) and 20 staff (mean [SD] age, 37 [8] years). Patient race and ethnicity (1 American Indian or Alaska Native [5%], 1 Asian and Pacific Islander [5%], 6 Black [30%], 6 Latine [30%], and 6 White [30%]) and primary SUD (7 patients with opioid and stimulant use disorder [35%]; 7 patients with stimulant use disorder [35%]; 3 patients with opioid use disorder [15%]; and 3 patients with alcohol use disorder [15%]) were diverse (Table 1). Of the 20 patient interviews, 17 (85%) were conducted in English. Staff represented diverse clinical services and roles. We identified 3 major themes related to perspectives on impact, with representative quotations summarized in Table 2: (1) expanded access to harm reduction*—*particularly for ethnically and racially minoritized populations; (2) built trust—participants viewed harm reduction as positively impacting the patient care experience and increasing engagement; and (3) catalyzed culture change—the program offered staff a framework for working with patients who planned to continue substance use, helping address stigma and increasing staff fulfillment. Based on these themes, we developed a model illustrating the perspectives of hospital-based harm reduction for patients and staff (Figure) and made recommendations based on the 3 themes (Table 3). We also identified several categories of program challenges, including hesitancy regarding regulations, lack of staff SUD education, remaining stigma, and the need for careful assessment of individualized goals.

**Table 1.** Characteristics of Patient and Staff Participating in Qualitative Interviews on the Impact of a Hospital-Based Harm Reduction Program

Characteristic	Participants, No. (%)
Patients (n = 20)	
Race and ethnicity	
American Indian or Alaska Native	1 (5)
Asian and Pacific Islander	1 (5)
Black	6 (30)
Latine	6 (30)
White	6 (30)
Primary substance use disorder	
Opioids and stimulants	7 (35)
Stimulants	7 (35)
Opioids	3 (15)
Alcohol	3 (15)
Age, mean (SD), y.	43 (13)
Primary language	
English	17 (85)
Spanish	3 (15)
Hospital staff (n = 20)	
Race and ethnicity	
Asian and Pacific Islander	5 (25)
Black	1 (5)
Latine	5 (25)
White	7 (35)
Multiracial	2 (10)
Age, mean (SD), y	37 (8)
Role	
Nurse	8 (40)
Clinician (physician or nurse practitioner)	5 (25)
Clinician trainee (resident or fellow)	4 (20)
ACT navigators and licensed vocational nurses	3 (15)
Department	
Non-ACT^a^	12 (60)
ACT	8 (40)

Abbreviation: ACT, addiction care team.

^a^ Non-ACT included emergency medicine, family medicine, internal medicine, and labor and delivery.

**Table 2.** Key Themes and Representative Brief Quotations From Patient and Staff Interviews on Their Perspectives of the Impact of a Hospital-Based Harm Reduction Program

Theme and subtheme	Quotation
Expanded access	
Increased access to safer use supplies	“I feel a big relief….With this city being a blatant crystal meth city, you should be a little bit more blatant to tell people, because people need their stuff…and people are really bad on their sharing needles….It’s too much risk.” –P18 (Black patient on learning how to use naloxone and safer injection supplies)
Provided access to harm reduction education and counseling	“There’s a lot of shame around it because I’m receiving it for having an overdose, but it’s good to have it, and I feel more supported and safe having it…knowing that I could talk to someone about it helped.”–P05 (Asian patient on having access to safer use supplies and naloxone)
Engaged patients with limited prior harm reduction exposure	“No sabía, y pues, me sirve, porque tantos amigos que tengo que puedo ayudar también…” [“I didn’t know, and well, it’s useful, because I have so many friends that I can help too…”] –P07 (Latine patient on learning how to use naloxone and fentanyl test strips for the first time)
Built trust	
Created a positive health care experience amidst pervasive stigma	“When I was offered harm reduction supplies…I didn’t feel like I was being judged harshly, but more understood. That my issues were being seen, not just as an addict…here to abuse the system or the help provided. I wasn’t seen as a perpetrator. The offer of supplies just really spoke a great deal about compassion.” –P13
Deepened patient engagement and relationships between patients and staff	“I think the biggest difference is how people interact with our team and how they feel during their admission, people all the time say…‘I’ve never been treated with as much empathy when it comes to my substance use.’” –S01 (ACT member)
Reduced likelihood of future delays in presentation for health care	“I would come back for sure a lot more easily. That time I overdosed…the way the EMTs were presenting it was like I wasn’t going to get any services like that, that it was going to just strap me down until I could get clean and try to force me into a program….It just definitely changed my outlook on coming to the hospital.” –P10
Catalyzed culture change	
It just makes sense	“We should be able to offer clean needles….Addiction is a hard thing. People die from this, and if we can prevent it and give the patient another chance to maybe seek help, that’s really important.” –S19 (surgical clinician)
Destigmatized care for those who continue to use substances	“In med school, it was kind of a black [and] white situation where…‘either you’re gonna stop using and we’re gonna give you all these medications to help you do that’ or ‘bye’…essentially…there is no in between. With the ACT team resource, you get the sense that we’re trying to meet patients where they’re at.” –S05 (internal medicine resident)
Increased staff fulfillment and countered burnout	“The culture that I came from, there was definitely that attitude around it. So, for me personally, it’s actually very refreshing to know that that’s something that’s available for anyone who comes in. And that they have a sense of being accepted or welcome where they are.”–S03 (medical and surgical nurse)

Abbreviations: ACT, addiction care team; EMT, emergency medical technician; P, patient; S, staff.

**Figure.**
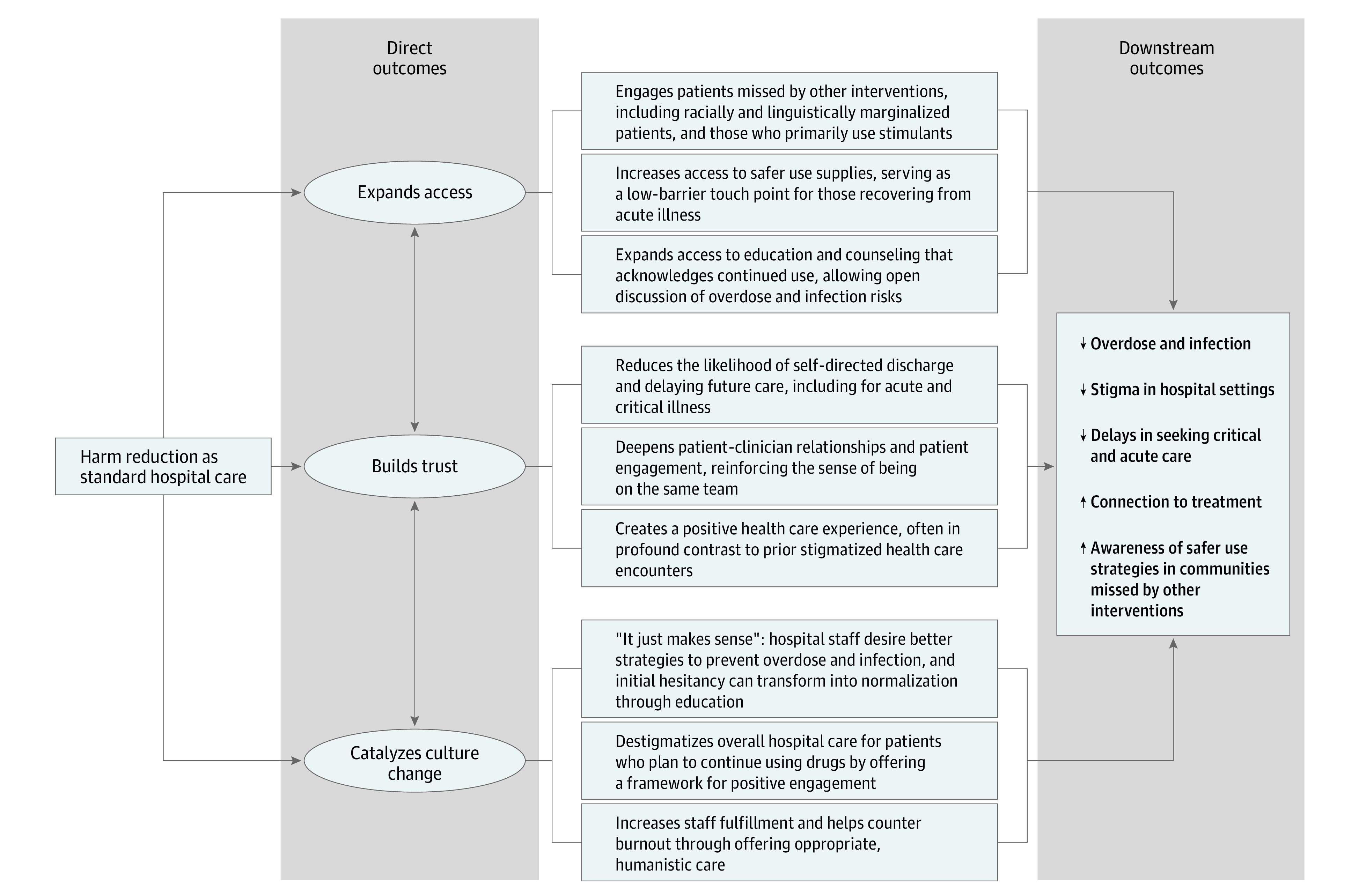
Conceptual Model of Harm Reduction as Part of Standard Hospital Care and Outcomes for Patients and Staff

**Table 3.** Summary of Recommendations by Theme Assessing the Perspectives on Impact of Hospital-Based Harm Reduction

Theme	Recommendations
Expanded access	Incorporate harm reduction education and supplies into standard hospital addiction care
	Invest in efforts to reach groups less exposed to harm reduction including Black, Latine and LEP patients with SUDs across substances
	Educate hospitalized patients who use substances about safer use and overdose prevention, including patients with stimulant use disorder
Built trust	Expand standard hospital care to include stigma-free engagement of all people who use substances, including those who plan to continue use
	Ask hospitalized patients about their goals for use and tailor care to support those goals, including discussion of strategies for reducing harm
	Offer harm reduction supplies, doing so can facilitate open conversations about continued use without judgment
Catalyzed culture change	Provide hospital staff with training on harm reduction as an evidence-based framework for engaging patients who plan to continue use
	Recognize that most hospital staff have received little formal training on SUDs and that hesitancy and stigma can shift over time through education
	Role model harm reduction principles for trainees and colleagues, doing so can powerfully reinforce new cultural norms
Addressing program challenges	Expand pathways allowing the provision of harm reduction services and clarify protections for health care workers providing such services
	Implement universal training on SUDs and harm reduction across the health professions
	Prioritize relationships and individualized patient goals when delivering harm reduction interventions, not solely supply delivery
	Partner with existing syringe service programs and other hospital harm reduction programs to learn best practices

Abbreviations: LEP, limited English proficiency; SUD, substance use disorder.

### Theme 1: Expanded Access to Harm Reduction

#### Increased Access to Safer Use Supplies

Patients and staff viewed access to safer use supplies at discharge as a convenient, low-barrier touch point. Some patients regularly accessed supplies at SSPs but appreciated the immediate availability at discharge, especially those recovering from injuries, older patients, and those with mobility impairments that made accessing SSPs postdischarge difficult. Others reported limited or no connection with harm reduction services due to lack of awareness, hesitancy about accessing SSPs, or chaotic use. Staff felt that the services reduced barriers. An ACT member explained, “It just felt really obvious and really strange that we tell people ‘this is where you can go’…but it’s like ok, you’re here, why don’t we just do this” (S10). A few staff expressed uncertainty about whether the intervention meaningfully increased access, whereas patients were more likely to emphasize the critical importance of access to supplies provided by the intervention, describing witnessing overdoses of loved ones and/or acquiring hepatitis C through reused supplies. One patient explained, “Giving the supplies is really, really helpful for the people…it really is” (P04).

#### Provided Access to Harm Reduction Education and Counseling

Patients and staff valued education on safer use strategies and counseling that acknowledged continued use as a possibility, highlighting the importance of discussing “actual drug use” (S11), such as route of administration, patterns of use, and reasons for use. Patients described worrying about fentanyl overdose and appreciated open discussions about safer use. Many patients were well-versed in harm reduction, while others reported learning new harm reduction strategies. These discussions increased empowerment and space for reflection, helping participants develop personal substance use goals. A Black patient explained, “Maybe this is God’s way to put [these conversations] in my path to get me to slow down, before I run into something out there on the street that’s harmful…I have the knowledge now of what I can do” (P01).

#### Engaged Patients With Limited Prior Exposure to Harm Reduction

The program engaged patients who reported limited prior harm reduction exposure, especially racially and ethnically minoritized patients and those who primarily used stimulants. Nearly all patients who reported learning new safer use strategies identified as racially or ethnically minortized individuals. Additionally, of patients who primarily used stimulants (all Black or Latine), many had personally experienced a prior opioid overdose and many reported learning new safer use strategies. Black and Latine participants expressed a need for access to harm reduction education and supplies in their communities. One patient described the need, “en las escuelas, en las iglesias…en la calle…en español, es importante” (“in schools, in churches…on the street… in Spanish, it’s important” [P14]). ACT staff also identified reaching patients with limited English proficiency (LEP) as a substantial impact, with many having limited knowledge of naloxone, including several Spanish-speaking patients we interviewed.

### Theme 2: Built Trust

#### Created a Positive Health Care Experience Amidst Pervasive Stigma

Patients commented on feeling cared for, and not feeling judged, as one of the program’s biggest impacts. Patients described how meaningful this was given long histories of stigmatization in health care settings. One patient explained, “They’re not just taking advantage of you and trying to just cold chuck you off…they’re trying [to] at least reach out, and for real help” (P18). Patients described extensive experiences with discrimination, poor care quality, and neglect in health care settings related to their SUD. While several staff endorsed tough love strategies as effective, nearly all patients viewed nonjudgmental services as critical to SUD care. Several patients described their hospitalization and receiving harm reduction services as their first positive hospital experience. One patient explained, “They really listened to me. And I haven’t had that for the longest, for years” (P04).

#### Deepened Patient Engagement and Relationships Between Patients and Staff

Staff echoed these sentiments, describing a shift in the care dynamic and increased engagement when they offered supplies or acknowledged patient goals for continued use. Staff reported that providing harm reduction services “builds a lot of trust” (S09) and “helps patients understand that we’re playing on the same team”(S02). Patients and staff viewed providing harm reduction services as an important means of implicitly communicating respect by acknowledging continued use without judgement. Staff also reported that by offering supplies, they had more conversations with patients about how they used substances; this increased staff comfort and skill in discussing substance use and fostering further trust and engagement.

#### Reduced Likelihood of Future Delays in Presentation for Health Care

Nearly all patients reported previously delaying hospital care or self-directing a discharge because of stigma, shame, and untreated withdrawal and pain, often with substantial health consequences. Patients reported that they would be less likely to delay future care, including addiction care, due to feeling respected and seen by ACT harm reduction culture. While many reported the most critical component to completing hospitalization was adequate opioid withdrawal treatment, patients also described how harm reduction–based approaches made hospitalization more bearable. A Latine patient explained, “They were not judgmental; they were not expecting me to say things that would lead me to failure…which has been my experience in the past. Can you imagine how many people or how many lives we could save if there was not this kind of barrier?”(P17).

### Theme 3: Catalyzing Culture Change

#### “It Just Makes Sense”

ACT staff described the program as having initially faced hesitancy from some non-ACT staff, with concerns including being unfamiliar with safer use supplies, personal liability, and in-hospital substance use. However, 2 years into program implementation, staff repeatedly reported that providing safer use supplies made sense to help reduce overdose and infection, including staff who expressed hesitancy about harm reduction principles. One nurse explained, “You know how expensive HIV meds are. We still end up providing them if they get positive, right? So, it’s better if they have the resources—the clean needle” (S15). Others described having limited prior exposure to harm reduction but becoming invested once exposed, explaining, “I wish that nurses can have more access to those types of supplies across California.” (S07). Patients also expressed surprise at receiving supplies at the hospital but found the services appropriate. One patient explained, “I was a bit surprised, but I thought it was really cool and forward thinking. Very accepting in a way. Rather than stigmatizing…we should treat it as an illness…and offer help where it’s required” (P13). ACT staff reported that over time and with proactive educational efforts, distributing harm reduction supplies had become normalized. Staff emphasized the importance of educating new staff about the program and addressing misconceptions, including through one-on-one conversations, teachings during nursing huddles, and resident didactics. When occasional hesitancy regarding the supplies arose, it was largely from new staff.

#### Destigmatized Care for Patients Who Continue to Use Substances

While not universal, staff described a tangible shift in hospital culture related to the program. Having a framework for working with patients who plan to continue use was novel and helped destigmatize care for those whose goal was not abstinence. Several staff remarked on tailoring care to meet patients’ stated substance use goals and decreased use of shame or coercion-based approaches. Non-ACT staff also spoke about decreased stigmatizing language when discussing patients, noting fewer sentiments like “‘oh, they’re just a crackhead…we’re gonna give less time and less compassion,’” and an emerging a culture of “there’s always something that can be done” (S05). This shift spread, fueled by ongoing educational ACT efforts, and role modeling by superiors and colleagues. Patients commented on how different the hospital culture felt compared with prior health care experiences. One patient explained, “I actually felt like I’m being helped rather than ridiculed” (P10).

#### Increased Staff Fulfillment and Helped Counter Burnout

Non-ACT staff described the program as “useful” (S14), “eye-opening, as well as just encouraging…” (S06), and “[giving]…more tools to help [patients] engage in their care” (S02). Staff described moral injury working amidst SUD-related stigma in health care settings and valued the program’s cultural impact. Staff described urgently wanting better strategies to address overdose and reported sometimes feeling powerless in supporting patients with SUDs. Staff felt the program helped address burnout because they felt they were providing more appropriate care. Some staff described harm reduction care as deeply fulfilling. An ACT member explained, “[It] brings back some humanity” (S11).

### Program Challenges

#### Concerns About Regulatory Ambiguity Creating Initial Obstacles

Although the program met regulatory requirements, staff concerns about regulations and legality initially fostered hesitancy. An ACT member explained, “Some of the challenges have been the stigma that we sometimes face, and also people fearing…the regulations…and the legal aspects, like is this legal for us to do?” (S09). Several nurses commented on liability being an ever-present concern for their nurse colleagues.

#### Lack of Education on SUDs Contributing to Ongoing Stigma and Hesitancy

All non-ACT staff supported providing harm reduction supplies, but several staff expressed hesitancies about harm reduction principles and concerns about enabling substance use. Such staff felt that their concerns would be addressed with better education about SUDs. They reported wanting to help patients with SUDs but feeling unsure how to do so effectively. Both hesitant and nonhesitant staff reported feeling frustrated with receiving little SUD education during their professional training, requesting harm reduction and SUD education broadly. One nurse explained, “I want to better understand how to support my patients, because not having been actually taught or supported in that learning formally, it’s a disservice, to our patients and to us” (S17).

#### Persistent Stigma and Discordant Care

While many described how harm reduction services led to destigmatization, culture change was not universal. Patients described positive experiences intermixed with negative ones. Several patients reported still experiencing stigma and were concerned about non-ACT staff knowing they had asked for supplies. A patient explained, “You don’t want to talk about [safer use supplies] in the hospital because…you’re going to be treated differently, they’re going to treat you as a drug user” (P17).

#### Carefully Assessing Patients’ Substance Use Goals

One patient whose goal was abstinence had a negative experience; the patient received supplies while asleep and was disturbed and triggered to return to use when finding them upon waking. The patient thought that supplies should be available to patients but emphasized the need for careful assessment of each patient’s goals, explaining, “See where their mind frame is at first, over just giving them a pipe…see if that’s what they want to do, don’t push it on ‘em” (P03).

## Discussion

To our knowledge, this is the first qualitative study in the US to examine patient and staff perspectives on hospital-based harm reduction. We found that patients and staff viewed these services as having expanded access, built trust, and catalyzed culture change while meeting important challenges. The model we developed using these results proposes how integration of harm reduction into standard hospital addiction care is associated with patient and staff outcomes, including engaging structurally marginalized patients missed by other interventions, reducing likelihood of self-directed discharge, and countering staff burnout.

Prior studies have focused on community-based harm reduction, and early studies are examining harm reduction in primary care.^29,30,31^ Our study builds on these findings, with patients and staff viewing hospitalization as an opportunity to offer overdose prevention education and harm reduction supplies, particularly for communities missed by existing access points.^32^ Additionally, participants reported that harm reduction–based care offered staff a tool to effectively engage patients, reducing stigma and building trust between people who use drugs and the health care system. We also found that efforts must be paired with careful assessment of patient goals and staff education.

This is, to our knowledge, the first study on hospital-based harm reduction to include extensive Black, Latine, and Spanish-speaking patient perspectives, which highlighted prominent disparities. While many harm reduction practices originate from racially and ethnically minoritized communities, our findings echo prior studies^33,34,35^ that revealed less access to harm reduction among Black, Latine, and LEP patients, and those primarily using stimulants. This is notable given structural racism’s role in the disproportionate criminalization of Black and Latine people who use drugs, racial and language-based health care disparities (including access to SUD treatment), and disproportionate overdose deaths among Black and Indigenous communities.^36,37,38^ Such participants felt harm reduction access was needed in their communities. The disparities that were revealed for participants using stimulants are also critical given the marked increase in overdose mortality in this group, particularly Black individuals, for whom cocaine-involved overdose mortality rates are now more than double that of White individuals, despite similar rates of substance use.^39,40^

Our findings on trust and culture change indicate that patients and staff view harm reduction services as improving care quality and engagement while decreasing staff stigma. We found that culture change is possible; while changes were initially met with hesitancy, they became progressively normalized over time. Staff found harm reduction to both be reasonable and increase professional fulfillment. Educating staff about harm reduction frameworks and role-modeling harm reduction principles to both trainees and colleagues helped reinforce new norms.

The program’s challenges also have meaningful implications. Despite endorsement from the health department and hospital leadership, some staff initially had concerns about liability from distributing supplies.^24^ Although use of federal funds to support SSPs has been allowed since 2015, some states ban such programs or limit them by requiring authorization by health departments.^41^ Clarifying regulations and the protections afforded to health care–based programs may help address concerns. We also found that lack of education contributed to stigma and hesitancy. Both hesitant and nonhesitant staff strongly desired harm reduction and SUD education, highlighting an opportunity for accrediting bodies and educational institutions.^42^ Finally, the negative experience of a patient mistakenly given supplies highlights the importance of prioritizing individualized patient goals.

We summarize recommendations based on these findings in Table 3. Given the toll of addiction nationwide and its prominence among hospitalized patients, US hospitals should consider incorporating harm reduction education and supplies as standard care, while comprehensively educating staff on SUDs.^43^ Hospitals can partner with SSPs and other hospital-based harm reduction programs to foster best practices and address challenges. Policymakers should consider pathways facilitating expanded provision of harm reduction services and clarify protections for health care workers providing services. Finally, investment in reaching groups less exposed to harm reduction. including Black, Latine, LEP individuals with SUDs, and those primarily using stimulants, is critical.

### Limitations

This study has limitations. We interviewed patients during hospitalization, and therefore could not evaluate postdischarge outcomes, including use of supplies, care engagement, and overdose rates. Our study was limited to a safety-net hospital in San Francisco, a city with many harm reduction resources. Local awareness of harm reduction may have contributed to staff acceptability of the program; notably, most staff reported limited prior exposure to harm reduction. Additionally, in regions with fewer services, hospital-based harm reduction may more substantially increase access.

## Conclusions

In this qualitative study of hospital-based harm reduction, we found that patients and staff viewed these services as increasing access for structurally marginalized populations, building trust, and destigmatizing care for patients with SUDs. Hospital leaders should consider strengthening training on harm reduction frameworks and partnering with local SSPs to provide services. Efforts serving Black, Latine, and LEP populations with SUDs, including those who use stimulants, are especially needed.

## Notes

Supplement 1. **eAppendix 1.** Patient and Staff Interview Guides **eAppendix 2.** Final Codebook

Supplement 2.
Data Sharing Statement
