# Supplementary material for: Patient and Staff Perspectives on the Impacts and Challenges of Hospital-Based Harm Reduction
Source: JAMA Netw Open. 2024 Feb 22;7(2):e240229. doi: 10.1001/jamanetworkopen.2024.0229 (PMC10884877; doi:10.1001/jamanetworkopen.2024.0229)
Supplement: Supplement 1. — eAppendix 1. Patient and Staff Interview Guides eAppendix 2. Final Codebook [file jamanetwopen-e240229-s001.pdf]

Supplemental Online Content

Fraimow-Wong L, Martín M, Thomas L. Patient and staff perspectives on the impacts and challenges of hospital-based harm reduction. *JAMA Netw Open*. 2024;7(2):e240229.
doi:10.1001/jamanetworkopen.2024.0229

eAppendix 1. Patient and Staff Interview Guides

eAppendix 2. Final Codebook

This supplemental material has been provided by the authors to give readers additional information about their work.

© 2024 Fraimow-Wong et al. *JAMA Network Open.*

eAppendix 1. Patient and Staff Interview Guides

Patient Guide – Harm Reduction Qualitative Study

Thank you so much for agreeing to participate in our interview about harm reduction services. There are two parts to the interview:

1. First, I will ask you questions about your experience with harm reduction.
2. Second, we will complete a 5-minute survey.

Thank you. Now we will begin the interview and start recording.

Start Recording. Ensure your phone is on speaker with microphone attached.

1. Can you tell me how you accessed harm reduction supplies and services before this hospitalization?
    a. What types of harm reduction supplies do you access or use?
    b. What experiences with harm reduction did you have before this hospitalization?
    c. What does harm reduction mean to you?
    d. What role does it have in your life?
    e. When did you first access harm reduction services (e.g., syringe access, naloxone, overdose prevention services)? (E.g. education, overdose prevention, naloxone, supplies)

2. What barriers have you experienced in accessing harm reduction services? (e.g. language accessibility, feeling uncomfortable, location/hours, physical accessibility, fear of asking for services, documentation concerns)
    a. If you do not regularly access harm reduction supplies and services, are there reasons why?
    b. Who in the community is not accessing harm reduction supplies or is unaware of overdose prevention strategies? What would be good ways to connect them to resources?

3. What substances were you actively using before being hospitalized?
    a. You may have been asked about this already, but I just want to make sure we can talk about this openly. What do you want to tell me about your drug use and what drugs you were using before being hospitalized?
    b. How does harm reduction play a role for *** (go through each substance used)?

4. Tell us what happened when you were offered harm reduction supplies during this hospitalization (e.g., when the ACT team came by and talked to you about drug use, receiving counseling on safer drug use)? *(During interview, be careful to tease out harm reduction services from ACT services in general)*
    a. What did you learn about harm reduction during this hospitalization?
    b. **Were you surprised to be offered harm reduction supplies in a hospital setting? What was surprising?
    c. How is that helpful to you?
    d. What did you not know?
    e. What specific supplies and education did you receive?
    f. How did this compare to other conversations you had with other hospital staff (doctors, nursing, physical therapy, etc.) about your substance use?
    g. How is the experience receiving the harm reduction supplies different than past healthcare experiences? Experiences here a few years ago?

5. Which harm reduction service did you find most helpful and why?
    a. Examples: teaching, community resources/education (SAS, never use alone), navigators, reviewing supplies (ask which supplies), language concordant providers, fentanyl test strips

© 2024 Fraimow-Wong et al. *JAMA Network Open.*

   b. How did the counseling and education feel?
   c. How relevant was the harm reduction counseling to your everyday experience with the use of drugs?
   d. How relevant were the supplies you received for your everyday experience with the use of drugs?
   e. How was the counseling in terms of the terms/language used? Was it easy to understand?

6. What was not/least helpful?

7. How do you plan to integrate/change your approach to harm reduction after discharge?
   a. How does receiving harm reduction services impact your thoughts on next steps with *** (substance of choice) use after leaving the hospital?
   b. How have your thoughts changed about other aspects of your use of drugs after leaving the hospital?

8. How did receiving harm reduction supplies impact how you received other care services during this hospitalization (e.g., related to addiction care, pain care, overall care)?
   a. **E.g., your relationship with your doctors (e.g., trust with health care workers)
   b. How did it feel to have possible plans for continued drug use accepted and responded to by your provider? Was it surprising?
   c. Your relationship with the nursing staff
   d. Your experience of pain or other symptoms during hospitalization
   e. Your pain treatment or any other treatment during hospitalization
   f. Did you delay coming to the hospital this time? If so, why?
   g. What brought you to the hospital?
   h. Did receiving harm reduction supplies impact your willingness and likelihood that you'll access care in the future and to stay in the hospital?

9. How did you feel your pain, withdrawals, and cravings were addressed in the hospital?
   a. Was it as respectful and adequate as it could have been?
   b. How did receiving harm reduction supplies impact your experiences of withdrawal, pain, and/or cravings?

10. Our hospital strives to ensure that our services feel inclusive for all patients and honor the different racial, ethnic and cultural backgrounds of those we serve. However, we know that this goal is not always achieved. Have you had any experiences in the hospital where you felt that your cultural or racial identity impacted the care you received related to harm reduction or drug use?
    a. Have you had any previous experiences where your racial identity impacted how you were able to access harm reduction services, either positively or negatively?
    b. Did concerns around discrimination, either based on race/ethnicity or other factors like language, gender identity documentation status cause you to delay care or impact your decision to come to the hospital?
    c. In an ideal world, how can we change harm reduction services in the hospital feel more inclusive and culturally relevant? (i.e. racially concordant providers, culturally accessible referrals, providers who speak my language etc)

11. How would you change the harm reduction services you received during hospitalization?
    a. What could have been better about the harm reduction services you received?
    b. What was missing from the harm reduction services?
    c. What should be continued in providing harm reduction services?

© 2024 Fraimow-Wong et al. *JAMA Network Open.*

12. What do you think about overdose prevention supplies like narcan and fentanyl test strips being more widely available to anyone at the hospital? For example if there was an education booth at the hospital entrance?
    a. What about safer use supplies like syringes or pipes?
    b. Or if the harm reduction supplies were available through the hospital, but there wasn't someone to talk to about them with?
    c. Are there other strategies you'd like to see to help people get supplies?

13. In a world where we could have a safe supply of drugs, what would this be like? What would we give out and what would they give in the hospital?
    a. How would having a safe supply impact your relationship with drugs? (i.e. some safe way of accessing and consuming drugs)
    b. If there was a safe place to use drugs in the hospital, how would that change your experience?

14. We're about to finish, is there anything else that you'd like to share about harm reduction the hospital that we haven't mentioned?

We've finished the questions. Thank you for sharing. Now I will stop the recorder and together we'll complete a 5-minute survey to finish.

STOP Recording

Now I'm going to ask you a couple of questions about you to help us understand the characteristics of patients we're serving with our harm reduction program.

What is your current age? ___

How do you identify your gender? _____

How do you identify your race? _______

Do you identify as Hispanic or Latino? Yes/no

Where are you going to be staying after leaving the hospital? _____

Are you open to having your quotes used for research, as in, presented in papers or at scientific meetings? The quotes would be anonymous and would not be linked back to you: **Yes/no**

© 2024 Fraimow-Wong et al. *JAMA Network Open.*

Hospital Staff Guide – Harm Reduction Qualitative Study

Thank you so much for agreeing to participate in our interview about harm reduction services. There are two parts to the interview:

1. First, I will ask you questions about your experience with harm reduction.
2. Second, we will complete a 5-minute survey.

Thank you. Now we will begin the interview and start recording.

Start Recording. Ensure your phone is on speaker with microphone attached.

1. Tell me what your role is in the hospital?

2. Before you took care of your patient on ***, what were your experiences and knowledge about harm reduction?
   a. How did you learn how about harm reduction?
   b. What experiences did you have with harm reduction outside of the hospital?
   c. What does harm reduction mean to you?
   d. What do you know about the evidence behind it?
   e. What does harm reduction currently mean to you?
   f. E.g. education, overdose prevention, naloxone, supplies, reduces infections

3. Can you describe what your experience was with having your patient receive harm reduction services during their hospitalization?
   a. What did you learn about harm reduction during this experience?
   b. What surprised you?
   c. What did you already know?

4. How do you think receiving harm reduction services impacted your patient's hospital care?
   a. How did it impact their pain care specifically?

5. How did ACT discussing harm reduction impact your views regarding:
   a. your patient
   b. **Caring for patients who use drugs in general
   c. Caring for patients who use drugs who are requiring pain treatment
   d. the addiction care team
   e. our hospital's approach to addiction/harm reduction policy
   f. your attitudes/approach towards patients with addiction/substance use disorders
   g. How does burnout / time pressure impacted your relationship with caring for people who use drugs?

6. How have your own views about harm reduction changed over time?
   a. What led to those changes?
   b. What about any change over time your own views about working with people who use drugs?
   c. How does burnout / time pressure impact your relationship with caring for people who use drugs?

7. What concerns do you have about providing harm reduction supplies?
   a. What are the potential negatives of providing harm reduction during hospitalization?
   b. What concerns or fears around causing harm do you have?
   c. How does providing harm reduction supplies impact your workflow?
   d. What challenges did you face with providing harm reduction? From the patient? From the staff? What about resources, skills, and time?
   e. How do you feel harm reduction has impacted how you view or treat patients who use drugs?

8. What would you change about how the hospital provides harm reduction services?
   a. What could the hospital do to make this intervention better?

© 2024 Fraimow-Wong et al. *JAMA Network Open.*

    b. What should the hospital continue doing?
    c. What could other parts of the hospital outside of the Addiction Care Team be doing better around harm reduction?

9. What was most helpful about ACT discussing harm reduction with your patient?
    a. Examples: teaching, community resources/education (SAS, never use alone), navigators, reviewing supplies (ask which supplies), language concordant providers, never use alone, fentanyl test strips

10. What was least helpful?

We've finished the questions. Thank you for sharing. I'm going to stop recording now and we can move onto the survey questions.

STOP Recording

Now I'm going to ask you a couple of questions about you to help us understand the characteristics of staff who completed the interview.

What is your current age? ___

How do you identify your gender? ____

How do you identify your race? ______

Do you identify as Hispanic or Latino? Yes/no

What is your current role in the hospital? _______

Are you open to having your quotes used for research, as in, presented in papers or at scientific meetings? The quotes would be anonymous and would not be linked back to you: **Yes/no**

© 2024 Fraimow-Wong et al. *JAMA Network Open.*

eAppendix 2. Final Codebook

Dedoose Codes Export for Project: ACT SHARP

"Just makes sense" Any mention of support for harm reduction supplies/interventions framed as reasonable, logical part of addiction care, with or without ascribing to broader harm reduction philosophies. May include discussions from participants deeply invested in harm reduction strategies but also includes those who have little familiarity with harm reduction and/or may express hesitation or stigma about drug use, people who use drugs, see the ultimate goal as abstinence.

Challenges Any mention of difficulties with implementing the intervention, including barriers and work arounds. May include difficulties with buy-in, training, logistics, approvals, language access etc as well as successful and unsuccessful attempts to overcome such challenges.

Time Pressure Any mention of the difficulty of providing hospital care including addiction care and harm reduction services as relates to not having enough time. May include feeling overworked, feeling unable to provide needed services due to lack of time, having too high of a patient load, ways ACT's harm reduction program relieves time pressure or exacerbates it.

Cravings Any mention of craving for substances, navigating cravings in the hospital, how cravings were addressed by hospital staff , impact on ability to complete hospital care, experiences with cravings outside the hospital, strategies to address cravings. May also include staff perspectives on working with patients with cravings.

Culture Change Includes any description of culture change around harm reduction, people who use drugs, drug use, treatment of substance use etc. May include institutional changes, changes in social norms, changes in processes, as well as personal change i.e transformation of stigma, improved confidence in working with people who use drugs etc.

Delays in accessing healthcare Any mention of history of delays or barriers accessing healthcare, including reasons for delay (i.e. stigma, untreated withdrawal, distrust etc), length of delay, impact of delay on health. Includes attempts to access healthcare that were unsuccessful e.g. "I tried to go to the ED for chest pain but left after I started going into withdrawal and they wouldn't help me." Also includes likelihood of delaying care in the future after the intervention.

Disparities Any mention of differences in healthcare quality or access based on race, gender, language, immigration status, insurance status etc including access to harm reduction and substance use care. Does not include experiences of discrimination outside of healthcare settings (see social determinants of health/social context of addiction).

Education and Counseling Any mention of experiences receiving or delivering harm reduction education or counseling as part of the intervention, including whether it was valuable, most/least content, topics discussed etc. Can include patient and provider perspectives, but focused specifically on patient education and counseling, does not include education efforts directed at staff (see Provider Education).

Empowerment/Dignity Any mention of empowerment, dignity, respect, autonomy etc as related to engaging with harm reduction, including both patient and provider perspectives.

Experiences Receiving and Delivering Supplies Inpatient experiences with receiving and providing the physical safer use supplies themselves, including patient and provider perspectives. May include experiences of surprise, opinions on utility of supplies, the types of supplies available, planned use for supplies, why supplies are useful/not useful.

Future Opportunities Patient and provider suggestions on how to improve harm reduction and substance use services in the future. Includes suggestions on how to reach more patients, improve the quality of services, suggestions of additional services etc. Does not include workarounds of things that are currently being done.

© 2024 Fraimow-Wong et al. *JAMA Network Open.*

Good Quotes Quotes that capture the essence of a theme or are worth revisiting when putting together the manuscript.

Hesitancy Any mention of hesitancy about harm reduction and safer use supplies, or non-abstinence based, non-punitive approaches to care. May include reasons why hesitant, concerns, as well as suggestions and strategies to ameliorate hesitancy.

In-hospital use Any mention of using drugs while in the hospital, including both patient and provider perspectives. May include experiences using drugs while hospitalized, reasons for use, repercussions. Also includes staff perspectives on in-hospital use, concerns, experiences caring for patients who use drugs while hospitalized.

Increased physical access Any mention of how the intervention increases physical access to harm reduction supplies and education, including patients perceptions of utility of having access to supplies in the hospital, impact of such access, provider perceptions around access to physical supplies.

Interprofessional communication Any mention of communication between staff of different types such as between providers, nurses and navigators. Includes challenges related to interprofessional communication, communication workflows and workflow breakdown, reasons communication is needed, strategies for success, frustrations, desires and opportunities etc.

Legal/Carceral System/Policing Any mention of experiences with the carceral system, policing, legal issues as related to drug use, including experiences inside and outside the hospitals. Includes experiences with mandatory treatment programs, positive and negative experiences with police presence in the hospital, fears of seeking care due to police presence.

Lessons learned Any discussion of lessons learned through the experience of providing harm reduction services and advice to other hospitals. May include perceived keys to success, regrets, what should be replicated, what was tried and did not work etc.

Miscellaneous Any notable content not well captured by another code

Models of care Any exploration of the different ways of providing harm reduction services in the hospital. For example, as a consult service, provision by primary teams, by navigators vs providers, tensions between specialty care vs. normalization into routine care. May include discussion of the impacts, drawbacks, and rationale for various models.

New patient exposure to harm reduction Any mention of exposure to new harm reduction information or supplies in the hospital by patients, including both patient and staff perceptions. May include experience of new exposure, perceived impact of new exposure, staff perceptions of what populations of patients are learning about new harm reduction resources for the first time in the hospital.

On-Site Champion Includes any description of the role of staff who serve to "champion" harm reduction, including both prior experiences with such a person, and when described as a suggestion, the impact of having such a person, strategies that a champion might use etc.

Overdose Experiences with overdose, either for self or a friend, family member or patient.

Stimulant Use and Overdose Any mention of stimulant use and overdose, either for self, friend, family member or patient.

Pain Any mention of pain, including histories of chronic pain, treatment of pain in the hospital and in prior healthcare settings, impact of untreated pain on ability to engage in care, relationship between pain and substance use, pain medication tolerance. Also includes provider perspectives on treating pain and withdrawal, dosing pain medications, “pain med seeking”, fears around pain medication and changes in perceptions around pain medication related to ACT.

Patient-provider relationship Any mention of the patient-provider relationship, including both patient and provider experiences of increased trust, feeling cared for, feeling seen, rapport. May also speak to impact of such experiences in the context of histories of stigmatization.

© 2024 Fraimow-Wong et al. *JAMA Network Open.*

Agitation/Tension Any mention of patient agitation or tension between staff and patients, including both patient and staff perspectives. Includes ways ACT's services are perceived to help relieve tension, deescalate conflict.

Feeling cared for Any mention of how ACT's harm reduction services facilitate patients feeling cared for and/or feeling seen, including significance of such feelings for overall care, relationships with staff, and relationships with the healthcare system. Can include contrasts with prior healthcare experiences.

Trust Any mention of trust between patients and staff, including increased trust as an impact of the intervention, ability to be open/honest about substance use and sensitive health concerns in general, ability to not be judged.

Patients getting missed Any mention of patient populations that might be missed by ACT's harm reduction services or by harm reduction services in the community, why patients get missed, missed opportunities to provide harm reduction.

Place matters Anything having to do with the uniqueness of providing harm reduction supplies specifically in the hospital or in healthcare settings. Conversely anything about the need for harm reduction in specific community spaces.

Prior experiences with substance use programs Any mention of prior patient experiences with substance use programs including inpatient and outpatient rehab, peer support groups (AA, NA), dual diagnosis programs, outpatient programs, medication-assisted treatment, drop-in services, Bridge clinic etc.

Prior Healthcare Experiences Any mention of prior experiences with the healthcare system, including hospital care, outpatient care, and substance use and treatment programs. Includes positive experiences and challenging experiences, such as those that felt stigmatizing or where the patients' needs were not met. Excludes experience harm reduction focused programs (See patient experiences with harm reduction).

Prior patient experiences with harm reduction Any experiences patients have previously had with harm reduction, or lack thereof. Includes descriptions of experiences, opinions about harm reduction, reasons why have not previously accessed harm reduction services.

Prior provider experiences with harm reduction Includes any provider exposure to harm reduction prior to the ACT-SHARP intervention, includes both descriptions of those experiences, prior perceptions of harm reduction, as well as statements expressing lack of prior exposure to harm reduction.

Provider Education Any mention of provider education and lack thereof, including efforts such as lectures, nursing huddle engagement, residents rotating through ACT, as well as descriptions of how substance use and harm reduction were covered or not covered during formal training.

Providers Learning from Patients Any description of a provider learning from patients, including changes in knowledge, perceptions, stigma, orientation to people who use drugs.

Training Opportunities Includes suggestions for what education is most needed, ways of delivering education, and what education has been most helpful and unhelpful.

Provider Fulfillment/Burnout Any mention of the felt experience of providers as it relates to providing or being able to provide harm reduction services including better ability to serve patients, reduced moral injury and burnout, increased empowerment/decreased helplessness etc. Also includes experiences with burnout, exhaustion, competing demands as relates to providing care and harm reduction services to people who use drugs.

Being able to do something Any mention of staff feelings of empowerment as relates to providing ham reduction services, including descriptions of having a way to engage with those continuing use, impact on overall care, feelings of self-efficacy.

Referrals to Resources Any mention of referrals to resources such as to mental health resources, peer recovery groups, treatment programs, as well as social resources such as food,

© 2024 Fraimow-Wong et al. *JAMA Network Open.*

housing, employment etc. May include perceived value of such resources, the experience of receiving referrals, lack of knowledge of referrals, barriers related to referrals, lack of appropriate treatment resources etc.

Safe consumption site Any description of experiences with safe consumptions sites, opinions about such sites. May also include opinions on safe supply.

Social Determinants of Health/Social Context of Substance Use Factors outside the hospital that may impact patients' health —perceived by providers or patients to impact their experiences with substances, including housing, income, neighborhood, employment status, citizenship status, etc, as well as any mention of structural oppression, trauma, racism, social networks, discrimination outside of healthcare settings.

Solutions/Workarounds Any description of ways that staff have successfully navigated obstacles, found workarounds, resolved challenges in delivering harm reduction services. Includes things that have been tried, excludes suggestions of possible solutions that have not been tried (see Future opportunities).

Space for Reflection/Autonomy Any discussion of the hospital as a space for reflection, autonomy, personal goals around substance use, the personal nature of recovery, respect for personal choice, the impact/importance of harm reduction as it relates to respecting personal choice and not feeling judged.

Stigma Any mention of stigma related to people who use drugs, drug use, utilization of healthcare services by people who use drugs. Also include stigma around harm reduction itself, distinguished from hesitancy with stigma being more intense, dogmatic, values based. Includes descriptions of the transformation of stigma, changes in provider perceptions around substance use, efforts to reduce stigma, and suggestions/opportunities for future efforts. Excludes patient experiences with stigma in healthcare (see prior healthcare experiences).

Substance use history Any mention of experiences with substances prior to hospitalization including drug(s) of choice, and relationships with substances.

Success Stories/Evidence Any mention of the desire for or importance of sharing success stories/evidence for harm reduction. Can include expressions of hesitancy, uncertainty, and curiosity about the evidence behind harm reduction, also includes any description of using success stories as a tool for addressing hesitancy.

Supplies Logistics Any mention of the logistics of getting supplies to patients at discharge, including challenges, workarounds, rationale for the current model and suggestions to improve. Can also include patient experiences with logistics of getting the medication at discharge.

Surprise Any mention of surprise about providing harm reduction services in the hospital, including both patient and staff perspectives.

The Emergency Department Any mention of the emergency department including patient and provider experiences, as well as mention of unique challenges and opportunities.

Withdrawal Any mention of withdrawal including quality of treatment of withdrawal in the hospital, impact on ability to complete hospital care, experiences with withdrawal outside the hospital, strategies to address withdrawal. May also include staff perspectives on working with patients in withdrawal, treating withdrawal.

© 2024 Fraimow-Wong et al. *JAMA Network Open.*
