# Supplementary material for: Patient and Staff Perspectives on the Impacts and Challenges of Hospital-Based Harm Reduction
Source: JAMA Netw Open. 2024 Feb 22;7(2):e240229. doi: 10.1001/jamanetworkopen.2024.0229 (PMC10884877; doi:10.1001/jamanetworkopen.2024.0229)
Supplement: Supplement 2. — Data Sharing Statement [file jamanetwopen-e240229-s002.pdf]

Data Sharing Statement

Fraimow-Wong. Patient and Staff Perspectives on the Impacts and Challenges of Hospital-Based Harm Reduction. *JAMA Netw Open*. Published February 22, 2024. doi:10.1001/jamanetworkopen.2024.0229

Data
Data available: No

Additional Information
Explanation for why data not available: We are not participating in data sharing due to the sensitive nature of the data and to protect study participant privacy.
